# A Fine-Tune Role of Mir-125a-5p on *Foxn1* During Age-Associated Changes in the Thymus

**DOI:** 10.14336/AD.2016.1109

**Published:** 2017-05-02

**Authors:** Minwen Xu, Olga Sizova, Liefeng Wang, Dong-Ming Su

**Affiliations:** ^1^First Affiliated Hospital, Gannan Medical University, Ganzhou 341000, China; ^2^Department of Biotechnology, Gannan Medical University, Ganzhou 341000, China; ^3^Institute of Molecular Medicine, University of North Texas Health Science Center, Fort Worth, TX 76107, USA

**Keywords:** Thymic aging, Foxn1 gene, microRNA-125a-5p, negative fine tune

## Abstract

Decline of transcription factor *FoxN1*, which predominantly regulates thymic epithelial cell (TEC) differentiation and homeostasis lifelong, is demonstrated to be casually related to age-related thymic involution. Whereas, a global role of microRNAs (miRNAs) has also been demonstrated to control and maintain TEC-constituting thymic microenvironment and to be changed in expression profile in the aged thymus. Therefore, it is urgently necessary to build knowledge regarding whether and which miRNAs regulate *FoxN1* gene in the aged thymus. We primarily compared changes in miRNA expression profile between young and aged murine TECs with *Mus musculus* miRBase-V20 arrays (containing 1892 unique probes), and clearly identified and validated that at least one miRNA, miR-125a-5p, was increased in aged thymus. Applying miR-125a-5p mimics was able to inhibit *FoxN1* 3′UTR luciferase activity in a 293T cell line and to suppress FoxN1 expression in murine TEC Z210 cells. Since a single miRNA can play a fine-tuning role to regulate expression of multiple genes and a single gene can be regulated by multiple miRNAs, our result adds a single miRNA, miR-125a-5p, into the panel of *FoxN1*-regulating miRNAs associated with thymic aging.

Age-related thymic involution is due to a disruption in homeostasis of thymic epithelial cells (TECs), which is regulated by transcription factor *FoxN1* gene [1-3]. Whereas, like most other genes, *FoxN1* gene should be regulated by multiple microRNAs (miRNAs). This is a currently accepted principle that miRNAs act as powerful modulators to regulate a wide array of biological processes in development, aging, cancer generation, and immunological responses in eukaryotic organisms [4-6]. Recently, ample evidence shows that a global role of miRNAs indeed directly regulates TEC’s function. This work was demonstrated by blocking miRNA developing pathways in TECs (with a *FoxN1* promoter-driven Cre-induced gene knockout approach) either from primary miRNAs to intermediate miRNAs (knocking out enzyme DGCR8) [7] or from intermediate miRNAs to mature miRNAs (knocking out enzyme Dicer) [8]. Additionally, in the naturally-aged thymic situation, miRNA expression profile was altered [9, 10], and single specific miRNAs, such as miR-18b and miR-518b, were reported to lead to down-regulation of *FOXN1* in differentiation from a stem cell line to epithelial lineage [11]. These emerging findings provide a regulatory axis from miRNAs’ fine-tuning *FoxN1* to age-related disruption of TEC homeostasis in thymic involution. However, based on the principle that a single miRNA can regulate multiple genes (usually several hundreds), while a single gene can be regulated by multiple miRNAs [4, 12], single miRNAs in the pool of *FoxN1*-regulating miRNAs associated with thymic aging still remains largely unknown.

*FoxN1* is an epithelial cell-autonomous gene, predominantly regulating development of TECs and skin keratinocytes [13]. Inborn *FoxN1* mutation results in thymic and hair follicle epithelial development failure [14-16] associated with primary immune deficiency [17-19] and hairless nude skin [20, 21], respectively. In postnatal life, FoxN1 is decreased with age, which is casually related to age-related thymic involution [1-3]. Mouse *FoxN1* gene contains nine exons with about one kilo-base pairs (1kb)-long 3′UTR (untranslated region), which is a potential region targeted by miRNAs for post-transcriptional regulation.

Mature miRNAs are a large group of conserved, single-stranded, around 22 nucleotide (nt)-long, non-coding RNAs that act as post-transcriptional regulators of gene expression by binding to a target coding mRNA through imperfect complementarity at multiple sites of 3′UTR of a gene, resulting in coding mRNA cleavage, translational repression, or chromatin modification [22-24] to interfere a gene expression [25], which is mediated by more than one miRNAs [4, 12]. It is demonstrated that although a single miRNA, miR-205, is highly and preferentially expressed in medullary TECs (mTECs), genetic knockout of it is not sufficient to induce a deficient phenotype [26].

In the current study, we primarily compared changes in miRNA expression profile between young and aged TECs with miRBase-V20 arrays (containing 1892 unique probes). We found there are 341 of them giving detectable signals in TEC from young and old mouse groups. Among the miRNAs with signal intensity over 500 units, we found 8 of them up-regulated and 4 of them down-regulated significantly in TECs from aged, compared to young, murine thymuses. Among those 8 up-regulated miRNAs, we identified that miR-125a-5p negatively fine-tunes FoxN1 expression by validation through a real-time RT-PCR, and confirmed by a *FoxN1* 3′UTR luciferase assay in a 293T cell line and a miR-125a-5p mimic to FoxN1 suppressive assay in a TEC line: Z210. Since a single miRNA can play a fine-tuning role in regulation of multiple gene expression, while a single gene can be regulated by multiple miRNAs [4, 12], a single miRNA, miR-125a-5p is not the sole miRNA in the panel of *FoxN1*-regulating miRNAs, but we indeed add a single miRNA into the panel associated with thymic aging.

## MATERIAL AND METHODS

### Mice and animal care

Thymus samples were freshly isolated from either young (2 months) or aged (19-21 months) mice (C57BL/6 genetic background). All animal experiments were performed according to the protocols approved by the Institutional Animal Care and Use Committee of the University of North Texas Health Science Center, in accordance with guidelines from the National Institutes of Health, USA.

### miRNA microarray and Real-time RT-PCR

Total RNAs for miRNA microarray were isolated from enriched TECs (based on our previously published enzymatic method [27]) of young and aged murine thymuses using QIAGEN miRNeasy Mini Kit (QIAGEN Cat#217004) according to the manufacturer’s instructions. The miRNA microarray assay was performed through a service by LC Sciences, LLC (www.lcsciences.com) with miRBase-V20 arrays containing 1892 unique probes. Total RNAs for real-time RT-PCRs from enriched TECs of freshly isolated murine thymuses or from TEC line Z210 were isolated with TRIzol reagent (Invitrogen) and reverse transcribed to cDNA with the SuperScriptIII cDNA kit (Invitrogen). Real-time RT-PCRs for miRNAs were performed with TaqMan® MicroRNA Assay kits of individual miRNA primers and probes (Applied Biosystems). Real-time RT-PCR of *FoxN1* primers and probe (TaqMan method) were as described previously [27]. Real-time RT-PCR was run on a Step-One-Plus thermal cycler system (Applied Biosystems). The microRNA results were internally normalized to U6snRNA control levels, while the relative expression levels of *FoxN1* mRNAs from the Z210 were internally normalized to GAPDH levels. The average ΔΔC_T_ value from multiple young animals was always arbitrarily set as 1.0 in each real-time PCR reaction.

### Bioinformatics prediction of the targets of miRNAs

The targets of the miRNAs were predicted using multiple databases, including the TargetScan (www.Targetscan.org), miRanda (www.microrna.org), microcosm Targets (www.ebi.ac.uk/enright-srv/microcosm), and Segal Lab of Computational Biology (http://genie.weizmann.ac.il/index.html). The predicted hits from each algorithm were sorted as per the scores.

**Figure 1.**
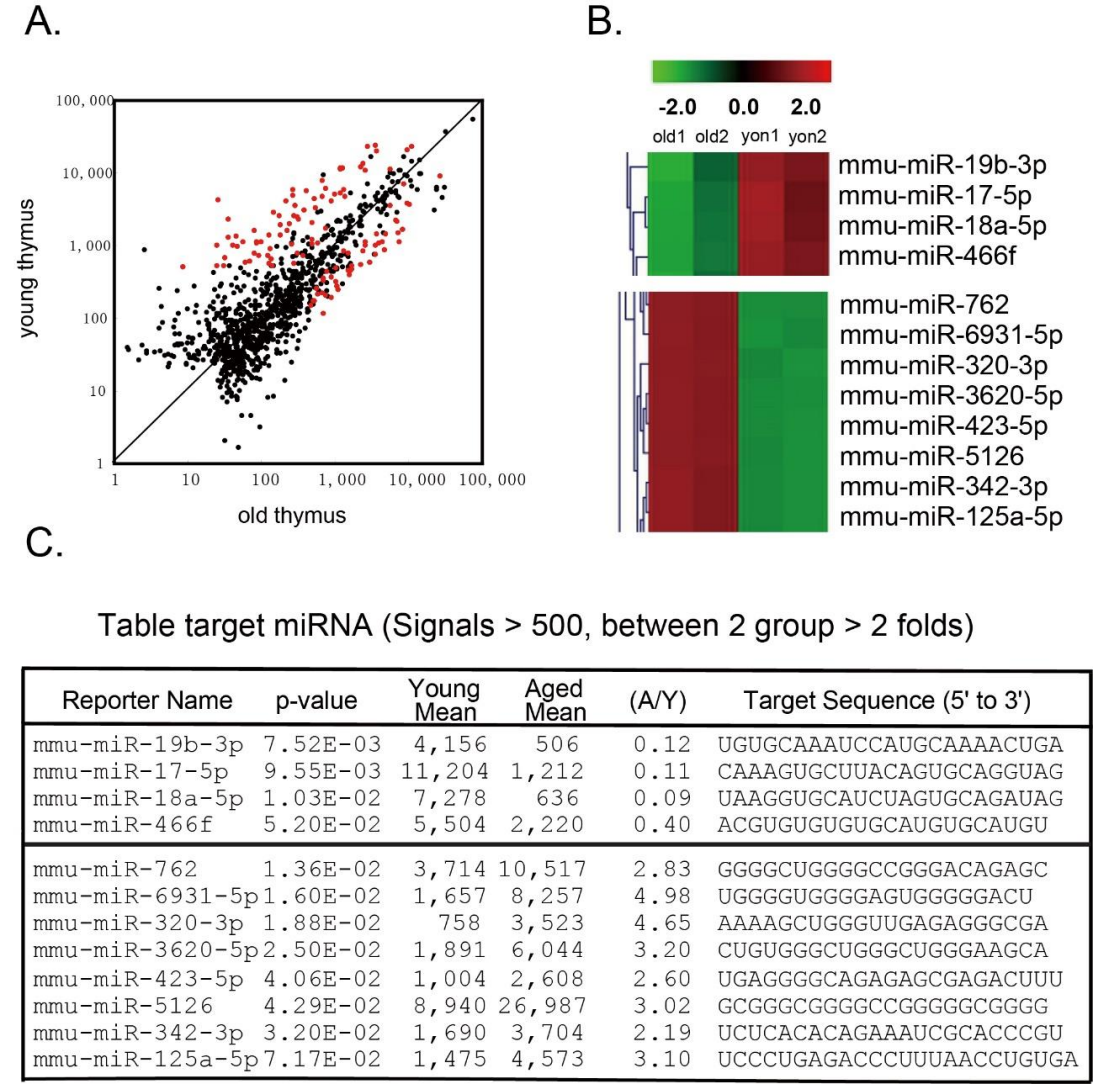
Microarray results of miRNAs expression from enriched TECs of young and old mice **(A)** A dot plot shows the positions of signals detectable miRNAs in TECs of both young and aged groups. Among these miRNAs there are 341 miRNAs (Red dots represent these miRNAs) with signal intensity over 500. **(B)** Heatmap shows 12 miRNAs which have signaling strength ≥ 500, decreased (≤ 2 folds) or increased (≥ 2 folds) in aged TECs compared to young TECs. **(C)** Detailed information about these 12 miRNAs.

### FoxN1 3′UTR plasmid construction and transfection

To generate a *FoxN1* 3′UTR luciferase reporter construct, we amplified a 1257-base pair (bp) DNA fragment consisting of the last 50bps of *FoxN1* coding region and 1203bps of the 3'UTR of *FoxN1* mRNA from a mouse bacterial artificial chromosome (BAC) clone RP24-137A13 DNA with PCR primers: Sense 5'-CCTG CCGTGTACCTCAGTC-3'; Antisense 5'-GGGACTG AAGGTCCCAAAAA-3'. This 1.257Kb DNA fragment of *FoxN1* containing the predicted target sites of miR-125a-5p was then subcloned into downstream of Gaussia luciferase (GLuc) that is driven by a phosphoglycerate kinase (PGK) promoter. Meanwhile, we also constructed plasmids with mutated target site on this DNA fragment to miR-125a-5p by replacing the seed sequences of miR-125a-5p (7-8 nucleotides at the 3' end of its target site, CTCAGGG to GAGTCAT) with scrambled sequences, which is termed m(364-387).

293T cells were maintained in RPMI 1640 supplemented with 1% penicillin/streptomycin and 10% fetal bovine serum. Lipofectamine 2000 (Invitrogen) was used in transient transfection of *FoxN1* 3′UTR luciferase reporter plasmids into 293T cells based on the manufacturer protocol. The medium was replaced with normal culture medium 6hr post transfection.

**Figure 2.**
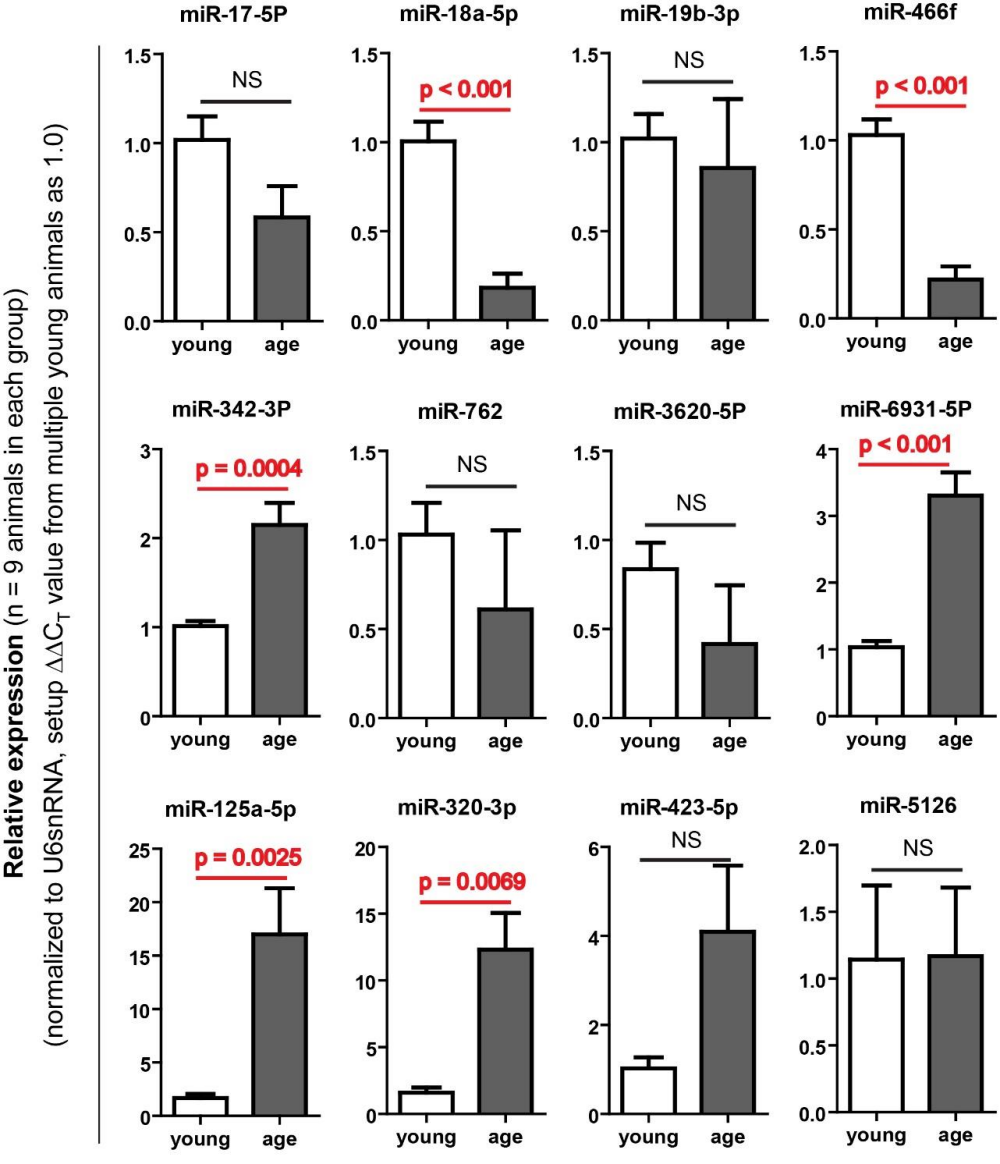
Validating 12 microRNA expression with Real-time RT-PCR The microRNA expression levels of miR-18a-5p, miR-466f, miR342-3p, miR-6931-5p, miR-125a-5p and miR-320-5p were consistent with the miRNA microarray results. The microRNA expression levels of miR-18a-5p and miR-466f were down-regulated in the aged thymus, while the microRNA expression levels of miR342-3p, miR-6931-5p, miR-125a-5p and miR-320-5p were up-regulated in the aged thymus. Data are presented as mean ± SEM (n = 9, i.e. Animal numbers in each group are 9 in three independent experiments). *p < 0.05, **p < 0.01 and ***p < 0.001, versus control (Student’s t-test). *p*-values are showed in each graph.

### Luciferase reporter assay

Each well of 293T cells cultured on a 12-well plate was transfected with 50ng of wild-type *FoxN1* 3′UTR luciferase reporter plasmids or m(364-387) mutated reporter plasmids, and miR-125a-5p mimics (with a various of concentrations ). Aliquots of medium from the transfected wells were collected at 24h, 48h, and 72h post transfection to measure luciferase activity. 50μl of the medium (diluted if necessary) was mixed with 100μl of substrate solution containing 0.5μg/ml of coelenterazine (CTZ), 200mM NaCl, 50mM Tris-HCl and 0.01% Triton X-100, at pH 8.7. The light emission was measured at a wavelength of 480 nm and normalized with the SEAP expression.

### miRNA mimics

Mmu-miR-125a-5p mimics was synthesized by GenePharma, Inc. (Shanghai, China), with the following sequences: miR-125a-5p, UCCCUGAGACCCUUUAA CCUGUGA; and negative control (miR-CON): UGACAACCUGGUAGAAAGAGACUUC.

### Statistics

Data are presented as mean±SEM. Statistical significance was analyzed by unpaired Student’s *t*-test and two-way ANOVA test. Differences were considered statistically significant at values of *p* < 0.05.

**Figure 3.**
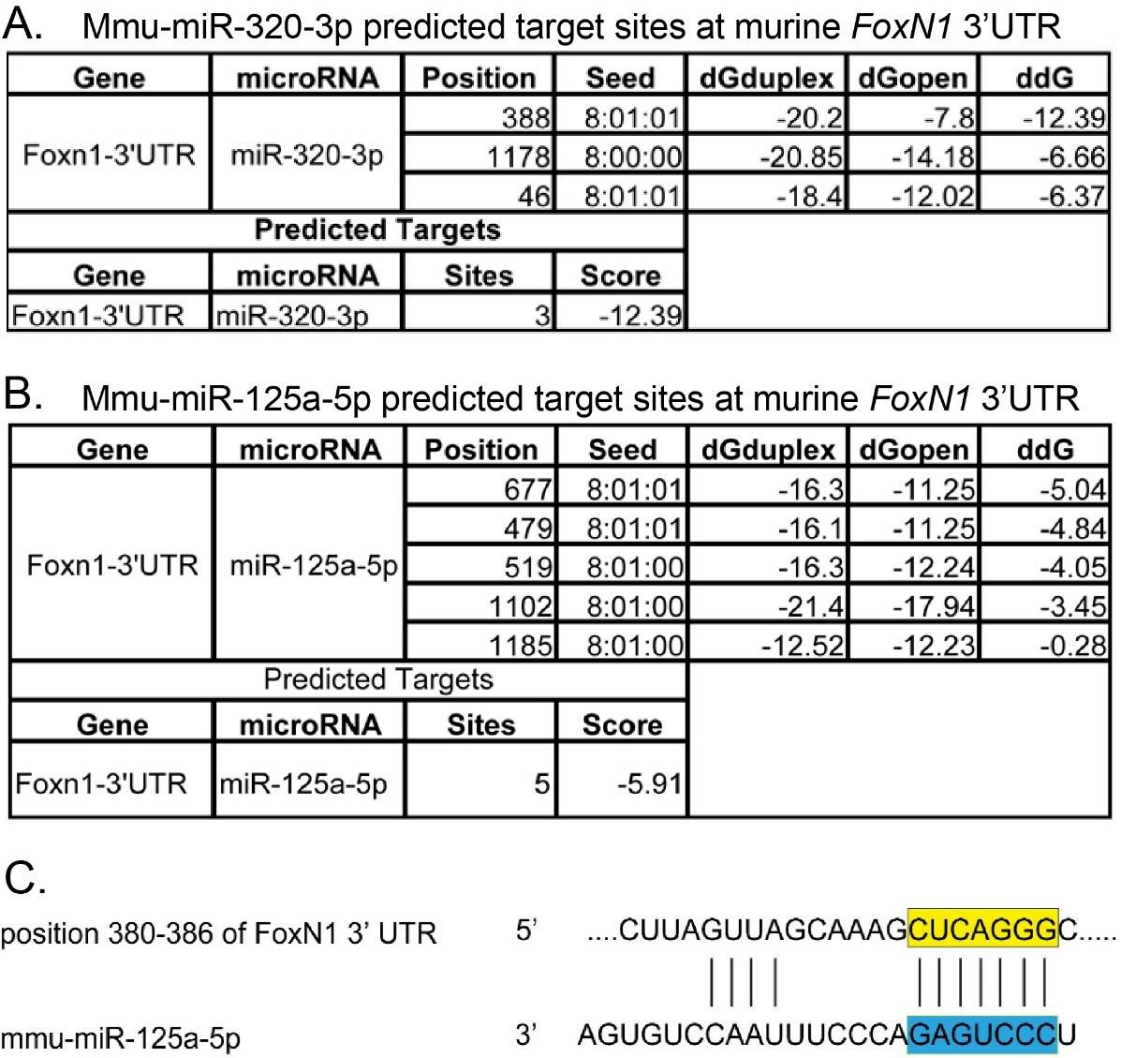
Computative analysis of predicted target sites of miR-320-3p and miR-125a-5p in *FoxN1* 3′UTR **(A)**. Result of scanning the *FoxN1* 3′UTR for miR-320-3p target sites based on databases. **(B)**. Result of scanning the *FoxN1* 3′UTR for miR-125a-5p target sites based on databases. **(C)**. Alignment of one predicted miR-125a-5p target site in the *FoxN1* 3′UTR.

## RESULTS

### Different expression profiles of miRNAs in TECs from young and aged murine thymuses

To screen age-related miRNAs in murine thymic epithelial cells (TECs), we assessed the miRNA expression profiles of total RNAs from enriched TECs isolated from the thymuses of 2-month-old and 21-months-old mice, respectively, with miRNA microarray (miRBase-V20 arrays containing 1892 unique probes, performed by LC Sciences). The results show that 341 miRNAs were detectably expressed in TECs with signal intensity over 500 in any of each (young or aged) or both (young and aged) groups (Red dots in Figure 1A). After future analysis by focusing on those with signaling strength ≥ 500, we found there are 4 miRNAs decreased (fold change ≤ -2.0) and 8 miRNAs increased (fold change ≥ 2.0) in aged TECs compared to young TECs. A hierarchical clustering/dendrogram analysis of these 12 miRNAs between 2 old (Old 1 & 2) and young (Young1 & 2) mice is shown in Figure 1B, and their detailed information is shown in Figure 1C, suggesting that TECs from young and old mice indeed have a certain of miRNA expression distinctly, and these twelve differently expressed miRNAs are potentially related to TEC-conducted thymic aging, associated with FoxN1 expression.

**Figure 4.**
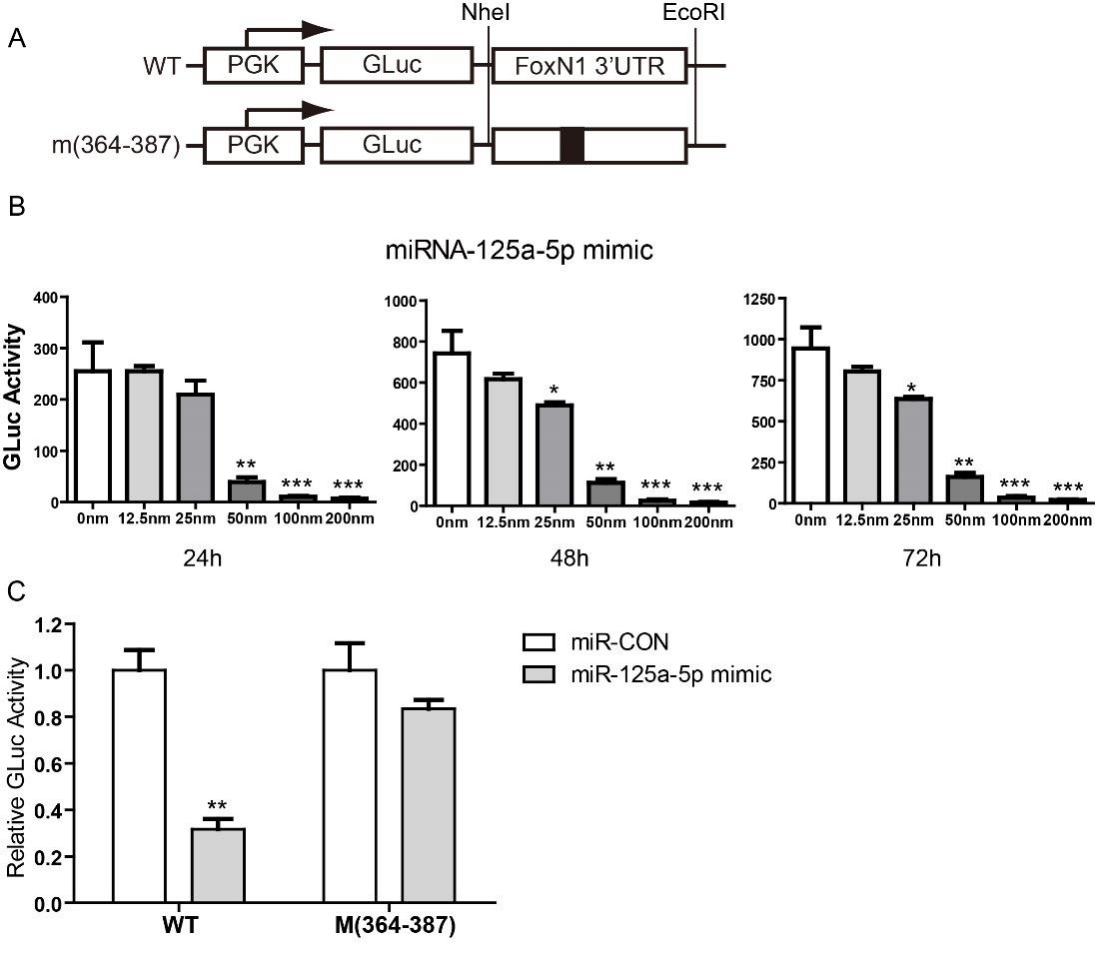
Results of miR125a-5p mimics inhibiting *FoxN1* 3′UTR luciferase reporter assay. (A) Schematic diagrams of construction of wild type (WT) *FoxN1* 3′UTR luciferase reporter (top panel), in which the last 50bp of *FoxN1* coding region and the first 1203 bps of *FoxN1* 3′UTR embracing the predicted miR125a-5p target sites is subcloned into downstream of GLuc driven by PGK promoter, and mutant *FoxN1* 3′UTR luciferase reporter (bottom panel), in which *FoxN1* 3′UTR at the first miR-125a-5p target site (364-387) was scrambled. (B) Results of inhibition to GLuc activity by miR-125a-5p mimics with a dose-dependent manner and time-course associated. GLuc activity was determined 24h, 48h, 72h post transfection and normalized by SEAP activity (see Materials and Methods for details). (C) One hundred nM of miR-CON (mimic negative control) or miR-125a-5p was co-transfected with 50ng of the wild type (WT) or mutant *FoxN1* 3′UTR luciferase reporter plasmids, and the SEAP-expressing plasmid. GLuc activity was measured 24h post transfection and normalized as described above. Data are presented as mean ± SEM (n = 9, i.e. nine independent experiments). *p < 0.05, **p < 0.01, and ***p < 0.001, versus control (two-way ANOVA test).

### Validation of expression of miRNAs in TECs potentially associated with thymic aging

To validate the results obtained from the microarray assay (Figure 1), we chose those screened 12 miRNAs to perform a more in-depth assay on their expression in TECs with more numbers of 2-month-old and 19-month-old mice by a real-time RT-PCR approach. Results are shown in Figure 2. In those 12 miRNAs, there were 6 miRNAs with statistically different expression, among which two (miR-18a-5p, miR-466f) were down-regulated (< -2.0 folds) in aged TECs which were consistent the result with ones in the microarray (Fig. 2 top panels), and four (miR-342-3p, miR-6931-5p, miR-125a-5p, miR320-5p) were up-regulated (> 2.0-fold), confirmed the trend in the microarray data (Fig. 2 middle and bottom panels). The real-time RT-PCR utilized more numbers of animals, which provide reliable biological standard deviation, confirm that microarray clues are informational.

### Predicting conserved target sites of potential miRNAs in the *FoxN1* 3′UTR

Since we focused on miRNAs with directly-inhibiting *FoxN1* role, we particularly aimed those up-regulated 4 miRNAs and predicted their potential target sites on *FoxN1* 3′UTR using the online target prediction programs [28] of TargetScan Microcosm Targets, and miRanda. We did not find targeting sites of miR-6931-5p and miR-342-3p in *FoxN1* 3′UTR, while we found that miR-320-3p and miR-125a-5p have 3 and 5 targeting sites in *FoxN1* 3′UTR, respectively (Fig. 3). Because miR-125a-5p has 5 targeting sites in *FoxN1* 3′UTR, in which nucleotide positions 364-387 were aligned with each other (as shown in Figure 3A top panel), miR-125a-5p attracts us. This clue suggests that miR-125a-5p has a higher potential to target *FoxN1* to play a negative fine-tune for inhibition of FoxN1 expression in aged thymus.

**Figure 5.**
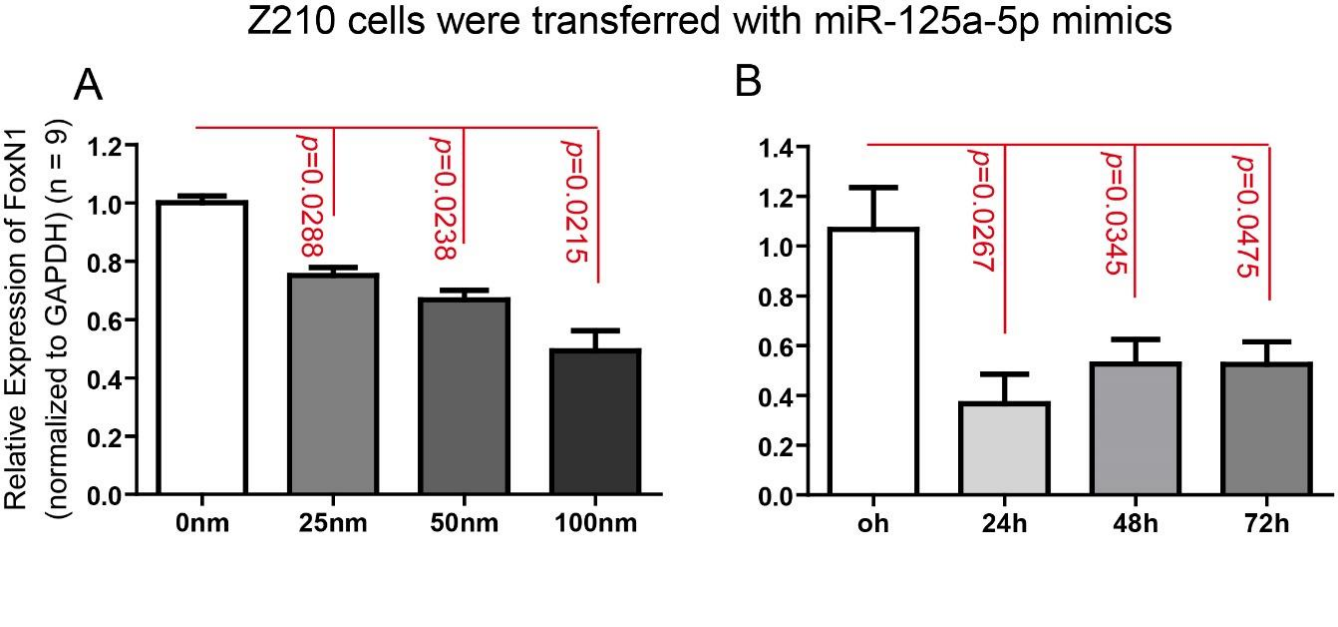
Inhibition of FoxN1 expression by miR-125a-5p mimics on thymic epithelial cells at dose-dependent and time-course manners. (A) TEC line Z210 cells were treated with increasing miR-125a-5p (0, 25, 50 and 100nM, compensated with miR-CON to 100nM, if necessary) for 48h. FoxN1 expression in Z210 cells was analyzed by Real-Time RT-PCR; (B) Z210 cells were treated with 100nM miR-125a-5p for 24h, 48h and 72h. FoxN1 expression in Z210 cells was analyzed by Real-Time RT-PCR. Data are presented as mean ± SEM (n = 9, i.e. nine independent experiments). *p < 0.05, **p < 0.01, and ***p < 0.001, versus control (two-way ANOVA test).

To further explore the potential role of miR-125a-5p in repressing FoxN1, we constructed a *FoxN1* 3′UTR luciferase reporter plasmid. A ~1.25Kb *FoxN1* 3′UTR DNA fragment [containing the last 50bps of *FoxN1* translated region, via PCR from a mouse bacterial artificial chromosome (BAC) clone RP24-137A13] was sub-cloned into downstream of Gaussia luciferase (Gluc) that is driven by PGK promoter (kindly provided by Dr. Sui) [29] (Figure 4). Meanwhile, we also made a mutant 3′UTR of *FoxN1* [a 7bp mutation on the first target site for miR-125a-5p, termed m(364 - 387)], as a control vector (Fig. 4A bottom panel), along with wide-type (WT) 3′UTR of *FoxN1* vector (Fig. 4A top penal). NIH293T cells were transfected with an either WT *FoxN1* 3′UTR luciferase reporter plasmid followed by adding synthetic mimics of miR-125a-5p with a final concentration of 0 - 200nM (Fig. 4B). A significant dose-dependent repression in the WT *FoxN1* 3′UTR luciferase reporter activity was observed at 24h, 48h and 72h post co-culture (Fig. 4B). In addition, a mutant *FoxN1* 3′UTR luciferase reporter plasmid m (364-387) (at 100nM) was also transfected into 293T cells. However, the suppression, seen in WT *FoxN1* 3′UTR transfection (Fig. 4B), could not be observed in the 24hr post co-culture compared with miR-CON (a negative control miRNA), which is a random sequence of a miRNA molecule that has been extensively tested in human cell lines and tissues not to produce identifiable effects on known miRNA function. (Fig. 4C). Taking together, the result that miR-125a-5p mimic was able to suppress post-transcription activity of WT 3′UTR of *FoxN1* at a dose-dependent manner, but could not suppress this activity of mutant 3′UTR of *FoxN1*, which indicates that miR-125a-5p specifically targets 3′UTR of *FoxN1*.

### *In vitro* effects of miR-125a-5p on *FoxN1* expression in a TEC line

Since FoxN1 is endogenously expressed in epithelial cells, to determine whether miR-125a-5p is able to fine-tune endogenous *FoxN1* expression, we transfected the synthetic miR-125a-5p mimics into murine TEC line Z210 (a murine thymic stromal cell line with a medullary phenotype, generated by Dr. Farr) [30] and observed inhibiting effects of miR-125a-5p on the TEC endogenous *FoxN1* expression with Real-time RT-PCR. As shown in Figure 5, increasing concentration of miR-125a-5p mimics (25, 50, 100nM) negatively regulates the expression of endogenous *FoxN1* in TECs at 48hrs post transfection (Fig. 5A). We further analysis the time-course inhibiting effect at a highest concentration of miR-125a-5p mimics (100 nM) and found that there is not a time-course change, but the inhibiting effect cannot be reversed even by 72hrs (Fig. 5B).

## DISCUSSION

The thymus is a unique organ to be responsible for T cell development and central tolerance establishment. Age-related thymic involution has a severe clinical outcome, which results not only in reduced output of naïve T cells but also in increased output of self-reactive T cells in the elderly [31]. The thymus consists of two major cellular components: thymocytes from hematopoietic origin and stromal cells, mostly thymic epithelial cells (TECs), from non-hematopoietic origin. TECs mainly constitute thymic microenvironment to support thymocyte development. However, TEC development and homeostasis are mainly regulated by an epithelial cell-autonomous transcription factor, *FoxN1* gene, through the *p63-FoxN1* regulatory axis [32]. Age-related thymic involution [33-35] has been demonstrated to be causally associated with decline of *FoxN1* [1-3]. However, decline of *FoxN1* with age could be mostly regulated by epigenetic alteration, since mounting evidence indicates that changes in many transcription factors are epigenetically regulated in the context of the aged microenvironment [36-40], where epigenetic modifications are associated with remodeling of chromatin structure to play a central role in controlling molecular expression [37-40] via alteration of transcriptional activity. Epigenetic regulation includes three major mechanisms at three layers: DNA methylation (DNA layer), histone modifications (chromatin layer), and non-coding microRNA expression (post-transcription layer). However, limited information is available on how changes in organism affect FoxN1 expression in the aged thymus. Our study suggests a new target at post-transcription level in the aged thymic context. We identified a potential *FoxN1* negative regulator, miR-125a-5p, which is able to fine-tune FoxN1 expression based on (1) increased miR-125a-5p expression in the aged thymuses, (2) an inhibiting effect on *FoxN1* 3′UTR luciferase reporter by transferring miR-125a-5p mimics, and (3) a dose-dependent decrease of endogenous FoxN1 expression in a TEC line by transferring miR-125a-5p mimics.

Regulation of TEC biological processes by miRNAs has been recognized in many context, particularly in TEC development [7, 8, 11]. *FoxN1* gene is critical in the regulation and maintenance of TEC biological function and it also controls postnatal TEC homeostasis. Therefore, miRNAs appear to be involved in the epigenetic regulation of TEC aging through repressing FoxN1 post-transcription in the aged thymus. However, the regulation of thymic aging by miRNAs’ suppressive function to *FoxN1* gene is the lack of evidence, while studying on miRNA profiles between young and aged thymus just began [9, 10]. It is well-known that a single gene can be regulated by multiple miRNAs. However, specific miRNAs on regulation of age-related decline of FoxN1 has been less well investigated. Although some miRNA candidates, such as miR-18b and miR-518b, were reported to lead to downregulation of *FOXN1* in stem cells [11], herein, we add one more candidate miR-125a-5p leading to downregulation of *FoxN1* in the aged thymus. A steady-state up-regulation of miR-125a-5p was also observed in 10- and 19-month-old murine thymuses by other group [9], which the possibility that miR-125a-5p is indeed involved in thymic aging associated with a decline of FoxN1. Because a single miRNA may only play a role for a fine-tune, we do not expect that totally abolishing FoxN1 expression can be achieved by knocking out or inhibiting single miR-125a-5p.

MiR-125 is a group of miRNA family, whose members include miR-125a-5p and miR-125b etc. MiR-125 play important roles in development and cell differentiation [41]. A significant down-regulation in miR-125a-5p and miR-125b was observed in malignant and aggressive breast cancer tissues [42]. An important role for miR-125a-5p in angiogenesis regulation via RTEF-1 was elucidated in aging mice [43]. Evidence also shows that miR-125a-5p may be as a potential tumor-suppressor in gastric cancer [44, 45], and acts as an inhibitor to epidermal growth factor receptor signaling to block migration and invasion of lung cancer cells [46]. These findings of mir-125a-5p as a suppressor of proliferation and migration in cancer cells may provide a clue that an increase of mir-125a-5p in the aged thymus can suppress thymic epithelial cell homeostasis during aging. Additionally, in non-cancer cells, miR-125a-5p also has a role in suppressing classical activation of macrophages while promoting alternative activation by targeting Kruppel-like transcription factor 13 (KLF13) [41], which negatively regulates inflammation associated with T cell activation. An important role for miR-125a-5p in angiogenesis regulation via RTEF-1 was elucidated in aging mice [43]. In a recent study, miR-125a-5p appears to cause inflammatory activation of THP-1 cells [47], and activates NF-κB activity in diffuse large B-cell lymphoma cells [48]. These findings may be reminiscent of increased inflammation in the aged thymus.

In conclusion, our finding suggests that miR-125a-5p plays a role as a negative regulator of FoxN1 in thymic involution during aging. Targeting miR-125a-5p in aged thymus should be one of therapeutic strategies to delay age-related thymic involution and/or improve thymic function in the elderly.

## References

[1] Sun L, Guo J, Brown R, Amagai T, Zhao Y, Su DM (2010). Declining expression of a single epithelial cell-autonomous gene accelerates age-related thymic involution. Aging Cell, 9: 347-357 20156205 10.1111/j.1474-9726.2010.00559.x PMC2894280

[2] Zook EC, Krishack PA, Zhang S, Zeleznik-Le NJ, Firulli AB, Witte PL, et al (2011). Overexpression of Foxn1 attenuates age-associated thymic involution and prevents the expansion of peripheral CD4 memory T cells. Blood, 118: 5723-5731 21908422 10.1182/blood-2011-03-342097 PMC3228493

[3] Bredenkamp N, Nowell CS, Blackburn CC (2014). Regeneration of the aged thymus by a single transcription factor. Development, 141: 1627-1637 24715454 10.1242/dev.103614 PMC3978836

[4] Bartel DP (2009). MicroRNAs: target recognition and regulatory functions. Cell, 136: 215-233 19167326 10.1016/j.cell.2009.01.002 PMC3794896

[5] Tomankova T, Petrek M, Gallo J, Kriegova E (2011). MicroRNAs: emerging regulators of immune-mediated diseases. Scand J Immunol10.1111/j.1365-3083.2011.02650.x 21988491

[6] Pritchard CC, Cheng HH, Tewari M (2012). MicroRNA profiling: approaches and considerations. Nat Rev Genet, 13: 358-369 22510765 10.1038/nrg3198 PMC4517822

[7] Khan IS, Taniguchi RT, Fasano KJ, Anderson MS, Jeker LT (2014). Canonical microRNAs in thymic epithelial cells promote central tolerance. Eur J Immunol, 44: 1313-1319 24515814 10.1002/eji.201344079 PMC4141217

[8] Zuklys S, Mayer CE, Zhanybekova S, Stefanski HE, Nusspaumer G, Gill J, et al (2012). MicroRNAs control the maintenance of thymic epithelia and their competence for T lineage commitment and thymocyte selection. J Immunol, 189: 3894-3904 22972926 10.4049/jimmunol.1200783 PMC3675876

[9] Ye Y, Li D, Ouyang D, Deng L, Zhang Y, Ma Y, et al (2014). MicroRNA expression in the aging mouse thymus. Gene, 547: 218-225 24956559 10.1016/j.gene.2014.06.039

[10] Guo Z, Chi F, Song Y, Wang C, Yu R, Wei T, et al (2013). Transcriptome analysis of murine thymic epithelial cells reveals ageassociated changes in microRNA expression. Int J Mol Med, 32: 835-842 23969555 10.3892/ijmm.2013.1471

[11] Kushwaha R, Thodima V, Tomishima MJ, Bosl GJ, Chaganti RS (2014). miR-18b and miR-518b Target FOXN1 during epithelial lineage differentiation in pluripotent cells. Stem cells and development, 23: 1149-1156 24383669 10.1089/scd.2013.0262

[12] Kim VN, Han J, Siomi MC (2009). Biogenesis of small RNAs in animals. Nat Rev Mol Cell Biol, 10: 126-139 19165215 10.1038/nrm2632

[13] Su DM, Navarre S, Oh WJ, Condie BG, Manley NR (2003). A domain of Foxn1 required for crosstalk-dependent thymic epithelial cell differentiation. Nat Immunol, 4: 1128-1135 14528302 10.1038/ni983

[14] Pantelouris EM (1968). Absence of thymus in a mouse mutant. Nature, 217: 370-371 5639157 10.1038/217370a0

[15] Pantelouris EM, Hair J (1970). Thymus dysgenesis in nude (nu nu) mice. J Embryol Exp Morphol, 24: 615-623 5493276

[16] Nehls M, Kyewski B, Messerle M, Waldschutz R, Schuddekopf K, Smith AJ, et al (1996). Two genetically separable steps in the differentiation of thymic epithelium. Science, 272: 886-889 8629026 10.1126/science.272.5263.886

[17] Frank J, Pignata C, Panteleyev AA, Prowse DM, Baden H, Weiner L, et al (1999). Exposing the human nude phenotype. Nature, 398: 473-474 10206641 10.1038/18997

[18] Cunningham-Rundles C, Ponda PP (2005). Molecular defects in T- and B-cell primary immunodeficiency diseases. Nat Rev Immunol, 5: 880-892 16261175 10.1038/nri1713

[19] Amorosi S, D'Armiento M, Calcagno G, Russo I, Adriani M, Christiano AM, et al (2008). FOXN1 homozygous mutation associated with anencephaly and severe neural tube defect in human athymic Nude/SCID fetus. Clin Genet, 73: 380-384 18339010 10.1111/j.1399-0004.2008.00977.x

[20] Schlake T (2001). The nude gene and the skin. Exp Dermatol, 10: 293-304 11589726 10.1034/j.1600-0625.2001.100501.x

[21] Mecklenburg L, Tychsen B, Paus R (2005). Learning from nudity: lessons from the nude phenotype. Exp Dermatol, 14: 797-810 16232301 10.1111/j.1600-0625.2005.00362.x

[22] Bartel DP (2004). MicroRNAs: genomics, biogenesis, mechanism, and function. Cell, 116: 281-297 14744438 10.1016/s0092-8674(04)00045-5

[23] He L, Hannon GJ (2004). MicroRNAs: small RNAs with a big role in gene regulation. Nat Rev Genet, 5: 522-531 15211354 10.1038/nrg1379

[24] Ambros V (2004). The functions of animal microRNAs. Nature, 431: 350-355 15372042 10.1038/nature02871

[25] Hobert O (2007). miRNAs play a tune. Cell, 131: 22-24 17923083 10.1016/j.cell.2007.09.031

[26] Khan IS, Park CY, Mavropoulos A, Shariat N, Pollack JL, Barczak AJ, et al (2015). Identification of MiR-205 As a MicroRNA That Is Highly Expressed in Medullary Thymic Epithelial Cells. PLoS One, 10: e0135440 26270036 10.1371/journal.pone.0135440 PMC4535774

[27] Cheng L, Guo J, Sun L, Fu J, Barnes PF, Metzger D, et al (2010). Postnatal tissue-specific disruption of transcription factor FoxN1 triggers acute thymic atrophy. J Biol Chem, 285: 5836-5847 19955175 10.1074/jbc.M109.072124 PMC2820809

[28] Kertesz M, Iovino N, Unnerstall U, Gaul U, Segal E (2007). The role of site accessibility in microRNA target recognition. Nat Genet, 39: 1278-1284 17893677 10.1038/ng2135

[29] Cao P, Deng Z, Wan M, Huang W, Cramer SD, Xu J, et al (2010). MicroRNA-101 negatively regulates Ezh2 and its expression is modulated by androgen receptor and HIF-1alpha/HIF-1beta. Mol Cancer, 9: 108 20478051 10.1186/1476-4598-9-108 PMC2881117

[30] Friend SL, Hosier S, Nelson A, Foxworthe D, Williams DE, Farr A (1994). A thymic stromal cell line supports in vitro development of surface IgM+ B cells and produces a novel growth factor affecting B and T lineage cells. Exp Hematol, 22: 321-328 8112430

[31] Coder B, Su DM (2015). Thymic involution beyond T-cell insufficiency. Oncotarget, 6: 21777-21778 26318588 10.18632/oncotarget.4970 PMC4673115

[32] Burnley P, Rahman M, Wang H, Zhang Z, Sun X, Zhuge Q, et al (2013). Role of the p63-FoxN1 regulatory axis in thymic epithelial cell homeostasis during aging. Cell Death Dis, 4: e932 24263106 10.1038/cddis.2013.460 PMC3847336

[33] Chidgey A, Dudakov J, Seach N, Boyd R (2007). Impact of niche aging on thymic regeneration and immune reconstitution. Semin Immunol, 19: 331-340 18024073 10.1016/j.smim.2007.10.006

[34] Fry TJ, Mackall CL (2002). Current concepts of thymic aging. Springer Semin Immunopathol, 24: 7-22 11974583 10.1007/s00281-001-0092-5

[35] Taub DD, Longo DL (2005). Insights into thymic aging and regeneration. Immunol Rev, 205: 72-93 15882346 10.1111/j.0105-2896.2005.00275.x

[36] Jaenisch R, Bird A (2003). Epigenetic regulation of gene expression: how the genome integrates intrinsic and environmental signals. Nat Genet, 33 Suppl: 245-254 12610534 10.1038/ng1089

[37] Berdasco M, Esteller M (2012). Hot topics in epigenetic mechanisms of aging: 2011. Aging Cell, 11: 181-186 22321768 10.1111/j.1474-9726.2012.00806.x PMC3490364

[38] Sedivy JM, Banumathy G, Adams PD (2008). Aging by epigenetics--a consequence of chromatin damage? Exp Cell Res, 314: 1909-1917 18423606 10.1016/j.yexcr.2008.02.023 PMC2464300

[39] Fraga MF (2009). Genetic and epigenetic regulation of aging. Curr Opin Immunol, 21: 446-453 19500963 10.1016/j.coi.2009.04.003

[40] Rando TA, Chang HY (2012). Aging, rejuvenation, and epigenetic reprogramming: resetting the aging clock. Cell, 148: 46-57 22265401 10.1016/j.cell.2012.01.003 PMC3336960

[41] Banerjee S, Cui H, Xie N, Tan Z, Yang S, Icyuz M, et al (2013). miR-125a-5p regulates differential activation of macrophages and inflammation. J Biol Chem, 288: 35428-35436 24151079 10.1074/jbc.M112.426866 PMC3853290

[42] Hsieh TH, Hsu CY, Tsai CF, Long CY, Chai CY, Hou MF, et al (2015). miR-125a-5p is a prognostic biomarker that targets HDAC4 to suppress breast tumorigenesis. Oncotarget, 6: 494-509 25504437 10.18632/oncotarget.2674 PMC4381610

[43] Che P, Liu J, Shan Z, Wu R, Yao C, Cui J, et al (2014). miR-125a-5p impairs endothelial cell angiogenesis in aging mice via RTEF-1 downregulation. Aging Cell, 13: 926-934 25059272 10.1111/acel.12252 PMC4331751

[44] Xu Y, Huang Z, Liu Y (2014). Reduced miR-125a-5p expression is associated with gastric carcinogenesis through the targeting of E2F3. Mol Med Rep, 10: 2601-2608 25231560 10.3892/mmr.2014.2567

[45] Song C, Wu G, Xiang A, Zhang Q, Li W, Yang G, et al (2015). Over-expression of miR-125a-5p inhibits proliferation in C2C12 myoblasts by targeting E2F3. Acta Biochim Biophys Sin (Shanghai), 47: 244-249 25733534 10.1093/abbs/gmv006

[46] Wang G, Mao W, Zheng S, Ye J (2009). Epidermal growth factor receptor-regulated miR-125a-5p--a metastatic inhibitor of lung cancer. FEBS J, 276: 5571-5578 19702827 10.1111/j.1742-4658.2009.07238.x PMC2776928

[47] Graff JW, Dickson AM, Clay G, McCaffrey AP, Wilson ME (2012). Identifying functional microRNAs in macrophages with polarized phenotypes. J Biol Chem, 287: 21816-21825 22549785 10.1074/jbc.M111.327031 PMC3381144

[48] Kim SW, Ramasamy K, Bouamar H, Lin AP, Jiang D, Aguiar RC (2012). MicroRNAs miR-125a and miR-125b constitutively activate the NF-kappaB pathway by targeting the tumor necrosis factor alpha-induced protein 3 (TNFAIP3, A20). Proc Natl Acad Sci U S A, 109: 7865-7870 22550173 10.1073/pnas.1200081109 PMC3356650

